# Bridging Divides or Widening Gaps? Nonprofit Organizations’ Efforts for Migrant Inclusion in Two Global Cities

**DOI:** 10.1177/08997640251387954

**Published:** 2025-11-11

**Authors:** Yan Long, Wei Luo, Berta Terzieva

**Affiliations:** 1 University of California, Berkeley, USA; 2 Peking University, Beijing, China; 3 Vienna University of Economics and Business, Austria

**Keywords:** diversity alignment, migrant inclusion, representative governance, interpersonal contact, state and market forces

## Abstract

Nonprofit organizations are often seen as critical nodes in shaping migrant experiences and supporting the inclusion and representation within host communities. In doing so, nonprofits encounter numerous obstacles. With this study, we explore the factors influencing how well nonprofits include, reach out to, and serve migrants relative to their local presence. We analyze organizational survey data from two distinct urban contexts: Vienna (Austria) and Shenzhen (China), each with unique institutional, political, and cultural backgrounds. Our findings suggest that interpersonal contact and representative governance in nonprofit organizations are closely linked to their (un)responsiveness to the needs of migrant populations. These results challenge the often-idealized perceptions of urban civic spaces and community interactions, underscoring the paradoxical mechanisms of inclusion and exclusion.

## Introduction

Recent scholarship highlights a “local turn” in migrant inclusion, with power shifting from the nation-state to municipal governance, expanding “downward, outwards and globally” (Oliver et al., 2020). Cities, offering opportunities and rich socioeconomic environments, have become crucial arenas for cultivating cosmopolitan democracy characterized by openness and inclusion and testing grounds for immigration policies (Schmidtke, 2014; Wickham, 2006; Yeoh, 2004). Migrant inclusion here emphasizes the host society’s efforts to acknowledge migrants, support their active participation, and foster their acceptance within social, cultural, and political life. It allows for diversity without necessarily requiring assimilation by focusing on equity and recognition. Nevertheless, systemic inequalities and exclusionary practices often undermine this vision of a genuinely inclusive society. Migrants, despite their contributions to urban prosperity, frequently face hostility and are seen as burdens on the welfare state (Savelkoul et al., 2017; Van Der Meer & Reeskens, 2021). They receive inadequate legal protections and social support, leading to more prolonged unemployment than natives (Veenman & Bijwaard, 2012), causing insecurities and instabilities that impede a truly inclusive urban society (Baban et al., 2017; Silvey & Parreñas, 2020; Swider, 2016).

Nonprofit organizations have been pivotal in mitigating challenges faced by immigrant minorities. By providing social services, influencing migration policies, and fostering political engagement (Bloemraad, 2006; Joassart-Marcelli, 2013; Varsanyi, 2010), these organizations fill gaps left by government and for-profit sector, which often lack the desire, capability, or public trust to meet the needs of immigrant minorities (Bloemraad et al., 2020; Hansmann, 1980; Weisbrod, 1977). This role extends beyond the contributions of migrant-focused organizations and migrant-specific initiatives. Empirical evidence consistently demonstrates that migrants’ participation in a wide array of civic activities can significantly enhance their social, cultural, and economic inclusion (Alexander et al., 2012; Greenspan et al., 2018; Handy & Greenspan, 2009). The density and diversity of a locale’s nonprofit organizations are strong indicators of its social capital and interpersonal trust (Putnam et al., 1993; Ruef & Kwon, 2016), level of civic engagement and political participation (Brandtner, 2022; Kim, 2015), and governmental capacity of implementing policies (Brodkin, 2006; Lecy & Van Slyke, 2013). At the organizational level, diverse leadership can enhance nonprofits’ ability to engage broader communities and empower racial and ethnic minorities, thus enabling organizations to better *walk the DEI talk* (e.g., Gooden et al., 2018; Hale & Finchum-Mason, 2023; LeRoux, 2009; Van Puyvelde et al., 2018). As such, nonprofits collectively exhibit a city’s infrastructural power (Clemens, 2020; Mann, 1987), essential for successful migrant inclusion.

Yet, despite their potential, nonprofits encounter numerous obstacles in effectively integrating migrants into host cities. Studies show that migrant engagement in nonprofit activities is significantly lower than that of the native-born population (Greenspan et al., 2018; Moya, 2005; Voicu & Şerban, 2012; Wang & Handy, 2014). Migrants face unique hurdles, such as residence status, language barriers, or unfamiliarity with local civic norms, which hinder their engagements with local civic spaces (Bloemraad et al., 2020). Indeed, social and cultural norms of participation in the country of origin and, even more so, in the host country shape individual propensity to participate in associational life (Voicu, 2014). For organizations that rely on word-of-mouth and informal recruitment strategies, reaching out to migrants poses considerable challenges (de Leon et al., 2009; Keeter et al., 2000). This difficulty is compounded when organizational leaders perceive the costs of pursuing equality initiatives to outweigh the benefits (Noon, 2007). In addition, growing elite orientations reduce nonprofits’ incentive and capacity to embrace inclusive practices (LeRoux & Medina, 2023; Salamon, 2012).

Notably, nonprofits respond to the influx of migrants in varied ways. Some become more inclusive, aligning themselves with evolving demographics, whereas others “hunker down” (Putnam, 2007, p. 149) and remain closed off, growing increasingly dissimilar from their surroundings (Alesina & La Ferrara, 2002). Research is limited to factors influencing a nonprofit’s ability to adapt to a diversifying environment and foster connections between migrants and local residents. Research needs to extend beyond migrant-focused organizations and initiatives to encompass the broader civic landscape. For example, consider a local dance club: does it see more migrants participating as the neighborhood’s migrant population grows, or does it remain exclusive to long-term natives? The key issue here is not the absolute number of migrants an organization serves, but how the organization, regardless of its primary activities, responds to the increasing presence of migrants in its community.

This paper investigates the relationships between nonprofits and their environments, highlighting three key aspects: the organizations’ connections with state and market forces, significantly shaping the urban environment; representative governance, assessing the internal composition and formal procedures of representation; and interpersonal contact, evaluating the social ties between organizations and their beneficiaries. We aim to understand the factors that condition the extent to which nonprofits include, reach, and serve migrants proportionately to their presence in the community—a concept we term “diversity alignment.” It refers to an organization’s ability to equitably include and provide services to both migrants and non-migrants, reflecting the respective proportions of these populations in the local community. We use “diversity” for brevity, but focus on migration diversity as the primary driving force shaping diversification in our study of urban environments.

Unlike prior research focusing on migrant-specific organizations, we explore a broader range of nonprofit organizations, with or without an explicit focus on migrant issues. Some scholars warn that migrant organizations might intensify intra-community bonds, hindering broader inclusion and potentially creating “parallel societies” (Vertovec & Wessendorf, 2010, p. 8). As discussed earlier, a narrow focus on migrant organizations can obscure the larger civil society’s role in nurturing a diverse and inclusive urban landscape. Our study hence adopts a more expansive approach, recognizing the varied needs of migrants utilizing civic services, spanning from tangible demands like child care, sports, civic education, and advocacy to intangible aspects such as social bonding and trust-building, thereby avoiding the assumption of “a false homogeneity of interest or identity” (Weldon, 2002, p. 1155).

Analyzing original data from a random sample survey in two diverse urban contexts—Vienna, Austria, and Shenzhen, China, we find that practices of representative governance and interpersonal contact shape organizational responsiveness to migrant populations in multifaceted ways. Urban migrants are generally underserved, with many nonprofit organizations supporting fewer migrants than their local population proportion. Contrary to existing studies, diverse staff and traditional representative mechanisms like elections do not necessarily lead to organizations that align better with the local diverse communities. While staff diversity can help organizations include and serve a beneficiary base that better reflects the community in its migrant composition, this effect is mediated by the intensity of personal interactions between staff and beneficiaries. Superficial interpersonal contact may even impede an organization’s responsiveness to its diverse environment. By examining an organization’s diversity alignment with its environment, we highlight mechanisms that can strengthen a nonprofit’s role in facilitating migrant inclusion.

## Theoretical Framework

### Diversity Alignment

The persistent diversity gap within nonprofit organizations, especially in upper echelons such as boards (LeRoux & Medina, 2023), and the overrepresentation of elite groups in nonprofit governance (Salamon, 2012; Weare et al., 2009) raise questions about these organizations’ legitimacy and efficacy in reflecting their constituents’ interests (Ostrower, 2007). Scholars have debated the extent to which organizational representation can generate positive outcomes for traditionally marginalized groups such as women and racial minorities (Weisinger et al., 2016).

This study examines the factors that shape nonprofits’ ability to equitably include, reach, and serve migrants in proportion to the migrants’ presence in the community. Focusing on migrant diversity in two urban contexts, we propose the term “diversity alignment” to capture how closely an organization’s outreach, governance, and service practices mirror its community’s demographic makeup. We use diversity alignment as a measurable indicator of migrant inclusion, which we define as migrants’ equitable access to and participation in the civic sphere—being seen, heard, and served in proportion to their presence, thereby underpinning the removal of barriers to recognition and acceptance in everyday institutional life. Diversity alignment, rather than the sheer number of migrants served, better serves as a diagnostic tool to assess to what degree nonprofit organizations are structured and responsive to reflect the demographic realities of their communities and provide a foundation for inclusion. In this way, we shift the analytical focus to organizational practices that enable or inhibit equitable civic participation. By adapting to changing demographics, the civic sector as a whole can better embrace migrants, regardless of each organization’s primary area of focus. As we show in the following, external state and market forces, nonprofits’ varying investment in representative governance, and interpersonal contact shape their different levels of responsiveness to migrant populations in their local communities.

### State and Market Forces

Two key external forces may influence nonprofits’ engagement with migrants. First, local urban policies and initiatives significantly shape migrant diversification and inclusion. City government policies, legal structures, and funding decisions can either facilitate or constrain nonprofits’ efforts to serve migrants in their communities (Light, 2012; Marrow, 2009). Second, the business sector’s perception of migrants as a labor force shapes the extent and nature of services provided to them (Guevarra, 2010; Sidhu & Rossi-Sackey, 2022). In global cities, the agenda of the local state and market often overlap, perpetuating inequalities affecting migrants (Juul, 2022; Long & Luo, 2022).

City-level policies often enable nonprofits to connect with migrant communities and promote their well-being, even when national policies are less accommodating (Chaudhary & Guarnizo, 2016; de Graauw & Vermeulen, 2016; Watson, 2024). Municipal government support, especially through public funding, proves essential for these organizations to effectively address migrants’ needs and assist their integration (de Graauw et al., 2013; Williamson, 2018). Such funding helps correct sectoral inequalities and increase the representation of underrepresented subgroups in voluntary organizations (Fyall, 2016; LeRoux & Medina, 2023; Salamon, 1995).

As economic hubs, cities attract businesses and affluent individuals whose contributions shape how nonprofit organizations support migrant needs. Private funding, recognizing migrants’ roles as workers and consumers, might enhance services targeted at this demographic. However, given the marginalized status of migrants, private donors may favor projects for specific communities, especially those with cultural or national connections (Hung, 2007; Lee, 2023; Weng & Lee, 2016), potentially steering civic efforts away from broader migrant services. Considering this complex interplay, we hypothesize that:

**Hypothesis 1 (H1):** Nonprofit organizations relying more on public funding are more likely to include, reach, and serve a beneficiary base reflective of the local urban population.
**Hypothesis 2 (H2):** Nonprofit organizations relying more on private funding are more likely to include, reach, and serve a beneficiary base reflective of the local urban population.

### Representative Governance

Previous research on representation (Guo & Musso, 2007; Pitkin, 1967) suggests that certain governance features can support and enhance organizations’ ability to make decisions that match a community’s interests and needs. According to existing theoretical frameworks, representation acts as a critical link connecting an organization to its constituents, embodying “the making present in some sense of something which is nevertheless not present literally or in fact” (Pitkin, 1967, pp. 8–9). We adopt the concept of representative governance to encompass a nonprofit organization’s structure and practices that recognize and address the needs, concerns, and preferences of its surrounding communities. We specifically focus on representation as “standing for,” including formal representation through electoral procedures and descriptive representation—mirroring the characteristics of the represented population.^1^

Empirical evidence on formal representation mechanisms is mixed. Some studies posit that mechanisms such as formal electoral procedures, enhance an organization’s ability to advocate for its constituents, with leaders selected by members more likely to align with diverse community interests (Leardini et al., 2017; Moggi et al., 2022; Phillips, 1998). Other studies contend that formal representation does not invariably enhance an organization’s responsiveness to constituent needs and might even hinder the inclusion of marginalized populations (Guo & Zhang, 2013; Jacobs & Skocpol, 2007; Levine, 2016). For instance, many nonprofit organizations restrict member participation to voting alone, excluding them from the nominating process (Barakso & Schaffner, 2008). Resource limitations, institutional hurdles, and a deficit in civic skills often impede the involvement of economically disadvantaged and less-educated individuals in formal elections. Despite these mixed findings, we hypothesize that:

**Hypothesis 3 (H3):** Nonprofit organizations using formal representation mechanisms for leadership selection are more likely to include, reach, and serve a beneficiary base that reflects the local urban population.

Research on descriptive representation suggests that diverse leadership enhances nonprofit organizations’ ability to advocate for racial and ethnic minorities, champion political interests, offer multicultural services, and engage the broader community, significantly bolstering their capacity to empower underrepresented groups (Gooden et al., 2018; LeRoux, 2009; Van Puyvelde et al., 2018; Yukich et al., 2020). Despite its insights, current scholarship focuses predominantly on boards of directors and executive personnel, overlooking other segments of the civic sector workforce. Rolf et al. (2022) show that nonprofit organizations in ethnically diverse communities tend to exhibit ethno-racial diversity at all organizational levels, highlighting a nuanced interplay between community composition and internal organizational diversity. Therefore, we hypothesize that:

**Hypothesis 4 (H4):** Nonprofit organizations with a staff composition reflecting the local population are more likely to include, reach, and serve a beneficiary base that mirrors the local urban population.

### Interpersonal Contact

In-group trust and out-group hostility are common across societies, with diversity often linked to reduced general trust and civic participation (Kwon et al., 2013; Putnam, 2007; Stolle et al., 2008). For example, the presence of ethnic minorities in neighborhoods has mixed effects on support for radical right parties (Rink et al., 2009; Schlueter & Scheepers, 2010). Natives often view in-group welfare recipients as more deserving than migrants (Van Der Meer & Reeskens, 2021). Importantly, these detrimental effects can be alleviated by fostering interactions among community members, indicating a dynamic interplay between community diversity, cross-group interactions, and social ties (Savelkoul et al., 2017; Stolle et al., 2013).

The effectiveness of interaction in reducing hostility toward migrants depends on the nature of engagement. Empirical evidence underscores that enduring, meaningful contact among diverse individuals facilitates social inclusion (Z. Liu et al., 2020). Regular conversations with minorities in local communities and frequent face-to-face interactions build trust through direct experience, potentially influencing broader social networks (Granovetter, 1973; Stolle, 1998; Stolle et al., 2008). In contrast, mere cohabitation or transient encounters often reinforce in-group bonds and may even entrench stereotypes (Fincher et al., 2014; Kwan, 2013).

Nonprofit organizations are prime facilitators for promoting deep and sustained interactions among diverse groups, helping migrants navigate urban societies and establish new social networks (L. Liu et al., 2018). These organizations provide spaces for intergroup contact among beneficiaries, staff, volunteers, and community members, allowing migrants to actively shape their urban experiences and create “place-specific spaces of rights and recognition” (Juul, 2022, p. 2372; Long & Luo, 2022). Deepening connections with beneficiaries can enhance an organization’s effectiveness, credibility, and longevity (Vermeulen et al., 2016). However, voluntary associations often mirror broader societal cleavages, forming clusters along ethnic, political, religious, or class lines (Paxton, 2002, 2007). Strong ties within these groups can lead to isolated organizations, perpetuating the adage that “birds of a feather flock together” (McPherson et al., 2001). Simultaneously, weaker ties, indicated by membership tenure and level of group involvement, may attract more trusting individuals (Stolle, 1998). Therefore, we hypothesize that:

**Hypothesis 5 (H5):** Nonprofit organizations whose staff engage more extensively with constituents (strong ties) are more likely to include, reach, and serve a beneficiary base reflective of the local urban population than those with less extensive engagement (weak ties).

## Research Context

This study examines the civic sectors of Vienna, an international migration hub in the West, and Shenzhen, a bustling epicenter of domestic migration in the East. Our deliberate pairing offers a unique lens to assess nonprofits’ diversity alignment, balancing the established narratives of city development in the Global North with the rising urbanization in the Global South. Adopting a relational comparison from comparative urban studies, we foreground migration as one of the defining features of urban contexts (Walton, 1982). This approach treats cities as interdependent analytical units rather than isolated entities, prompting us to consider how insights from one inform understanding of another (Ward, 2010). Unlike studies confined to the same country or region, which assume shared sociopolitical and cultural experiences, our analysis expands the narrative of urban migration and examines organizational dynamics to provide an alternative to conventional cause-and-effect models.

Vienna and Shenzhen face similar challenges regarding migrant populations, relying on nonprofit organizations for migrant inclusion and prioritizing municipal decision-making. Despite these similarities, they exhibit maximized contrasts in their historical, cultural, and political contexts and how state and market forces shape their respective migration patterns (Mahoney, 2012). This duality helps us explore how nonprofit organizations with similar characteristics demonstrate varying degrees of responsiveness to a diversifying environment.

Our study focuses on migrants with a native language other than German in Vienna and residents without hukou status in Shenzhen. Vienna’s population is 35.2% first-generation international migrants (Statistik Austria, 2022), predominantly non-German speakers, with language skills being a significant gatekeeper to social and economic inclusion (Holzinger, 2020; Wodak & Boukala, 2015). In Shenzhen, over 12 million of its 17.5 million residents are domestic migrants (National Bureau of Statistics of China, 2021), and the permanent household registration system (“hukou”) institutionalizes urban-rural disparities, barring migrant workers from essential services, resulting in a “race-like status” for migrants in cities (Han, 2010, p. 593; Liang, 2016).

Both cities present similar challenges for migrants, including restrictive policies, limited opportunities, and discrimination. Migrants face marginalization in the labor market, lower wages, and increased poverty (Chen, 2011; Muckenhuber et al., 2022). They also contend with social stigmas and are often viewed as safety risks, with language proficiency providing minimal defense against bias (Krzyżanowski & Wodak, 2017; L. Liu et al., 2018; Weichselbaumer & Schuster, 2021). Exclusion permeates social benefits and health care, highlighting the pervasive nature of their marginalization (Kohlenberger et al., 2019; Liang, 2016; Samkange-Zeeb et al., 2020; Verwiebe et al., 2019).

Nonprofit organizations in both cities are vital catalysts for migrant inclusion, forging strong connections with government agencies to craft and execute government policies for migrants. Influenced by political parties, the Catholic Church, and the unique Austrian corporatism, Vienna’s nonprofit organizations rely heavily on public funds to support migrants (Maier et al., 2022; Neumayr et al., 2017; Pennerstorfer & Schneider, 2022). These organizations work closely with Vienna’s Department for Integration and Diversity (MA17, the former nonprofit *Vienna Integration Fund*^2^) to implement migrant-related policies and deliver services, promoting community cohesion and instilling a sense of solidarity in times of migration tensions (Hofmann et al., 2019; Schnelzer et al., 2023). Similarly, Shenzhen’s civic sector, with over 9,000 registered organizations (Chinese Academy of Social Sciences, 2017), engages in a state-society interplay within a neo-corporatist framework (Peng, 2022). Despite China’s authoritarian regime, Shenzhen adopts a regulatory approach similar to Vienna’s, utilizing service contracts and market mechanisms to guide and shape nonprofit organizations, granting nonprofits some autonomy in policymaking and advocacy (Su et al., 2022).

Vienna and Shenzhen demonstrate the crucial role of cities in navigating and sometimes countering broader national trends. Cities are primarily responsible for managing migrants’ legal statuses and enacting policies that balance their inclusion and exclusion (Juul, 2022; Schmidtke, 2014; Van Breugel, 2020). Vienna’s social democratic governance and its diversity policy aimed at fostering social cohesion contrast sharply with Austria’s restrictive national migration policies and the rise of populist right-wing parties (Hadj Abdou & Ruedin, 2022; Meyer & Rosenberger, 2015). Similarly, Shenzhen’s unique characteristics preclude it from representing China’s national migration policies. In the early 2000s, an influx of migrant workers led to the bourgeoning of grassroots labor organizations and prompted liberal-oriented city officials to implement reforms like community-based unionism, a practice unimaginable elsewhere in China (Lin, 2023). More recently, despite efforts to reduce migrant labor while enhancing its cosmopolitan image, a few education nonprofits continue to unite diverse individuals and facilitate migrant social inclusion (Long & Luo, 2022).

In sum, despite the seemingly unconventional pairing, Vienna and Shenzhen’s similar challenges with migrant populations, the significance of nonprofit organizations, and the central role of municipal governance provide a unique cross-continental comparison that enriches empirical understanding and advances theoretical development in urban migrant inclusion.

## Methods

### Survey Methodology

The data for this paper stem from two organization-level surveys conducted between 2018 and 2020 as part of a transnational research collaboration. These surveys targeted top leaders (e.g., executive directors, presidents) from randomly selected nonprofit organizations listed in the Shenzhen Municipal Bureau of Civil Affairs,^3^ the Austrian Register of Associations, and the Austrian Business Register.^4^ A standardized questionnaire was used in both cities to minimize bias, covering leadership, constituents, operations, mission, funding, and collaboration. This paper concentrates on responses related to the organizations’ demographic characteristics, operational practices such as leadership selection, beneficiary involvement in decision-making, staff interaction with beneficiaries, funding sources, and other organizational aspects.

In Vienna, 358 out of 712 nonprofit organizations completed the survey, yielding a 50.3% response rate. In Shenzhen, 203 out of 510 organizations responded, marking a 40% response rate. Data-collection strategies were adapted to each locale’s unique cultural and political contexts. In Vienna, hand-signed postal invitations were followed by phone and email reminders, with most responses received online and around 20% via phone or in-person interviews. In Shenzhen, in-person surveys and structured interviews were conducted at the organizations’ locations, averaging 2.5 hours, with all interviews recorded and transcribed.

Descriptive statistics for both samples (provided in the online Supplemental Materials; see Tables S1–S3) show that, in both cities, the largest share of organizations operates in the field of culture and recreation (24% in Shenzhen, and 45% in Vienna, with sports associations being particularly common), followed by social services (Shenzhen: 22%, Vienna: 12%) and business and professional associations (Shenzhen: 22% and Vienna: 10%). Vienna’s sample is representative in terms of field of activity and geography (Terzieva et al., 2024). Nonprofits in Vienna are older on average (32.1 years) than those in Shenzhen (7.4 years), reflecting the city’s longer institutional history. Membership organizations are also more common in Vienna (87%) than in Shenzhen (60%). Finally, and central to this study, both cities show discrepancies between the migrant share in the population (Shenzhen: 70%, Vienna: 24%) and their representation among nonprofit staff (Shenzhen: 38%, Vienna: 10%) and beneficiaries (Shenzhen: 43%, Vienna: 18%).

### Measures

#### Dependent Variable

**Figure 1.**
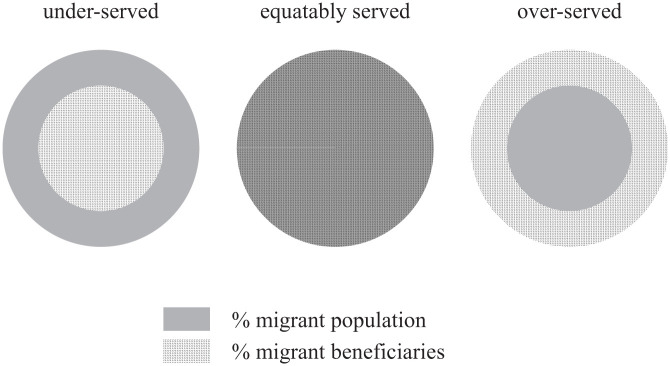
Illustration of Diversity Alignment.

With this study, we aim to better understand the factors that influence the likelihood of nonprofits to equitably include, reach, and serve a beneficiary base reflective of the local urban population—an organization’s diversity alignment. As illustrated in Figure 1, an organization may be *under-*serving migrants if it serves few migrants in a migrant-rich neighborhood and, conversely, *over-*serving if it serves many migrants in a homogeneous neighborhood, although the latter is rare.

To measure diversity alignment, we constructed the dependent variable by calculating the difference in proportion between the share of non-German/-hukou beneficiaries within the nonprofit organization and the share of the non-German/-hukou population in the organization’s primary geographic area of operation. This approach is commonly used in existing literature measuring representation (Hale & Finchum-Mason, 2023; LeRoux, 2009).

diversity alignment = % migrant beneficiaries – % migrant population

#### Independent Variables

Our independent variables are constructed based on survey questions, including descriptive workforce representation, formal representation, weak and strong workforce-beneficiary contact, and public and private funding. We control for organizational size, membership presence, and primary field of activity, reflecting the urban resource environment. Table 1 provides a comprehensive summary of these measurements and associated descriptive statistics.

**Table 1.** Description of Variables and Measures (Vienna/Shenzhen).

Variable/Measure	N	Min	Max	Mean	*SD*	Description/coding
**Dependent variable**						
Diversity alignment	267/133	−0.42/−0.86	0.85/0.41	−0.07/−0.32	0.21/0.30	Difference in proportion between the share of non-German/-hukou beneficiaries within the nonprofit organization and the share of the non-German/-hukou population in the organization’s primary geographic area of operation (similar to Hale & Finchum-Mason, 2023). The variable could hypothetically range from −1 to 1. A value closer to 1 or −1 indicates, respectively, overrepresentation or underrepresentation of non-German/-hukou beneficiaries, a value of 0 indicates exact representation between non-German/-hukou composition of beneficiaries and composition of non-German/-hukou population.
**Independent variables**						
Descriptive representation of workforce	267/133	−0.45/−0.78	0.85/0.41	−0.14/−0.33	0.18/0.28	Difference in proportion between the share of non-German/-hukou workforce (paid or volunteer) and the share of non-German/-hukou population in the organization’s primary geographic area of operation (similar to Hale & Finchum-Mason, 2023; LeRoux, 2009). The variable could hypothetically range from -1 to 1. A value closer to 1 or -1 indicates, respectively, over- or underrepresentation of non-German/-hukou workforce, a value of 0 indicates exact representation between non-German/-hukou composition of workforce and composition of non-German/-hukou population.
Formal representation	267/133	0/0	1/1	0.30/0.31	0.46/0.46	Dichotomous variable indicating who could elect the organization’s executive directors. In Shenzhen, respondents were directly asked a yes/no question about executive director elections, while in Vienna, we constructed an indicator based on beneficiary or member involvement in the elections.
Weak workforce-beneficiary contact	267/133	0/0.2	1/1	0.75/0.84	0.26/0.22	Additive scale comprises two items, each measured from 1 to 5, with 1 being practically no one/seldom and 5 being practically everyone/always. Respondents were asked to assess how well the workforce knows and engages with the organization’s beneficiaries: they recognize each other by name; they greet each other on the street. The variable was normalized to range from 0 to 1.
Strong workforce-beneficiary contact	267/133	0.13/0.2	1/1	0.47/0.61	0.22/0.25	Additive scale comprises two items, each measured from 1 to 5, with 1 being practically no one/seldom and 5 being practically everyone/always. Respondents were asked to assess how well the workforce knows and engages with the organization’s beneficiaries: they spend time together outside of the organization’s setting or activities; they give each other advice about topics unrelated to the organization’s activities; they participate in each other’s life events (e.g., attend birthdays, weddings). The variable was normalized to range from 0 to 1.
Public funding	267/133	0/0	1/1	0.16/0.37	0.28/0.37	Proportion of nonprofit organization’s total budget coming from public sources.
Private funding	267/133	0/0	1/1	0.22/0.18	0.32/0.30	Proportion of nonprofit organization’s total budget coming from private sources
**Control variables**						
Organizational size	267/133	0/0	17.73/17.22	9.44/12.88	3.01/2.27	Measured using the natural logarithm of the annual total revenue of the organization (e.g., Kim, 2017).
Membership organization	267/133	0/0	1/1	0.91/0.63	0.29/0.48	Dichotomous variable indicating whether the organization has members. Variable takes on the value of 0 (no) and 1 (yes).
Primary field of activity	267/133	1/1	3/3	-	-	Aggregated version of the International Classification of Nonprofit Organizations, ICNPO (Salamon & Anheier, 1996). Organizations were manually assigned to one of the ICNPO categories by the researchers, based on the organization’s name and mission. The aggregated version comprises 1 = recreation (sports, culture and arts, other recreational activities, and religion); 2 = service (social services, education and research, health, and housing); and 3 = advocacy (international, law and advocacy, environmental protection, professional unions, and others not else classified).

### Analytical Strategies

**Figure 2.**
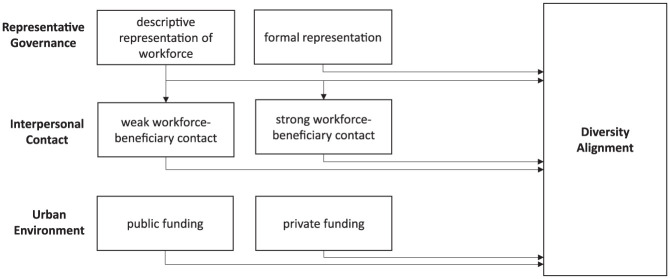
Factors Contributing to Nonprofit Organizations’ Diversity Alignment.

Generalized linear regression (GLM) with a normal (Gaussian) distribution was used to estimate the effects of various organizational characteristics on the diversity alignment of nonprofit organizations. We employed a stepwise approach to observe how additional specifications affect dependency relationships in the model. First, we included the hypothesized predictors: descriptive representation of workforce, formal representation, weak and strong workforce-beneficiary contact, and public and private funding. Next, we added control variables, including size, membership, and primary field of activity. Finally, we included interaction effects between the descriptive representation of the workforce and the degree of workforce-beneficiary contact (weak and strong). Figure 2 summarizes our theoretical framework and empirical model.

To ensure robustness, we used bias-corrected bootstrap *p*-values and confidence intervals based on 5,000 bootstrap samples. Bivariate correlations of the estimated variables are shown for each city in Tables S4 and S5 in the Supplemental Materials, indicating moderate to high correlations for some explanatory variables. A variance inflation factor (VIF) analysis was conducted to assess multicollinearity. In the initial model stages, adjusted generalized VIF (GVIF) scores ranged from 1.02 to 1.36. In the final model with interaction effects, adjusted GVIF scores ranged from 1.03 to 5.24. All values were below the standard threshold of 10, indicating that multicollinearity is not a significant concern in the models.

## Findings

**Figure 3.**
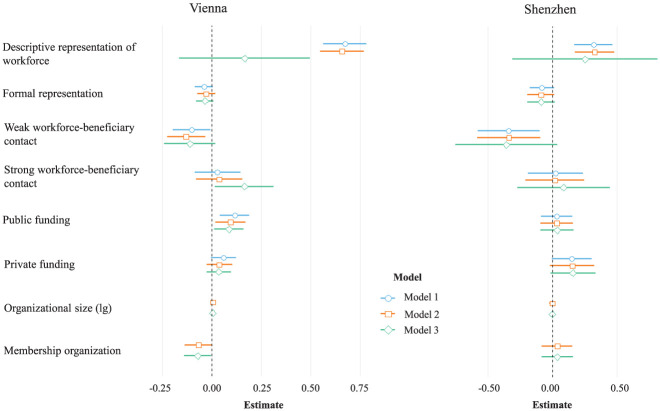
Coefficient Plot of Diversity Alignment by Various Organizational and Environmental Factors, Illustrating Main Findings of Table 2.

The model results are displayed in Figure 3 and Table 2. In both Vienna and Shenzhen, the main predictor of an organization’s diversity alignment is the *descriptive representation of its workforce* (Vienna: *b* = .672, *p* < .001; Shenzhen: *b* = .320, *p* < .001). This effect remained significant after including control variables, indicating its robustness across different organization sizes, membership statuses, and activity areas (Vienna: *b* = .657, *p* < .001; Shenzhen: *b* = .329, *p* < .001). This finding supports H4, suggesting that nonprofit organizations with a workforce reflecting the immigrant composition of their surrounding communities are more likely to reach and serve a beneficiary base that resembles that population.

**Table 2.** Results for the Hypothesized GLM Regression Model.

	Diversity alignment					
	Vienna			Shenzhen		
	Step 1	Step 2	Step 3	Step 1	Step 2	Step 3
***Descriptive representation of workforce***	0.672***	0.657***	0.165	0.320***	0.329***	0.254
	(0.056)	(0.056)	(0.168)	(0.075)	(0.079)	(0.289)
**Formal *representation***	–0.040*	–0.029	–0.035*	–0.080	–0.088	–0.087
	(0.023)	(0.023)	(0.023)	(0.048)	(0.054)	(0.055)
**Weak workforce-beneficiary contact**	–0.103*	–0.131**	–0.112	–0.339**	–0.338**	–0.358
	(0.049)	(0.049)	(0.067)	(0.123)	(0.124)	(0.202)
**Strong workforce-beneficiary contact**	0.029	0.036	0.164*	0.025	0.020	0.088
	(0.060)	(0.059)	(0.076)	(0.110)	(0.116)	(0.185)
**Public funding**	0.114*	0.094*	0.087	0.033	0.034	0.037
(% total income)	(0.038)	(0.039)	(0.038)	(0.062)	(0.064)	(0.065)
**Private funding**	0.059	0.037	0.035	0.150	0.155	0.159
(% total income)	(0.033)	(0.033)	(0.032)	(0.080)	(0.088)	(0.089)
**Budget (lg)**		0.004	0.004		–0.001	–0.002
		(0.003)	(0.003)		(0.011)	(0.011)
**Membership organization**		–0.068	–0.072		0.038	0.039
		(0.036)	(0.035)		(0.061)	(0.062)
**Primary field of activity (ref. Recreation)**						
Service		0.014	0.018		0.044	0.046
		(0.023)	(0.022)		(0.063)	(0.063)
Advocacy		–0.058*	–0.056*		0.019	0.026
		(0.028)	(0.028)		(0.060)	(0.062)
**Descriptive representation of workforce × Weak workforce-beneficiary contact**			0.075			–0.050
			(0.301)			(0.446)
**Descriptive representation of workforce × Strong workforce-beneficiary contact**			0.859**			0.188
			(0.328)			(0.393)
**Constant**	0.074	0.120	0.048	0.096	0.068	0.046
	(0.034)	(0.057)	(0.059)	(0.097)	(0.157)	(0.181)
**R** ^2^	0.399	0.426	0.459	0.247	0.252	0.254
**N**	267	267	267	133	133	133

*Note.* Unstandardized coefficients (b) with standard errors (SE).

*** *p* < .001; **p < .01; *p < .05. Significance is based on bootstrap *p*-values.

Contrary to expectations, formal representation (i.e., procedural mechanisms of selecting organizational leaders such as formal elections) showed a small negative association with diversity alignment in Vienna and it was not statistically significant in Shenzhen, providing no support for H1. Instead, there is an indication of a potential adverse effect.

In both cities, the extent of *workforce-beneficiary contact* demonstrated a robust negative effect for weak ties between staff members and beneficiaries (Vienna: *b* = –.131, *p* < .01; Shenzhen: *b* = –.338, *p* < .01), whereas strong ties were not statistically significant, suggesting weak ties may harm diversity alignment, supporting H5.

Private funding was not significantly associated with diversity alignment. Public funding showed a small positive effect in Vienna (*b* = .094, *p* < .05), which remained significant after adding control variables, providing partial support for H1 but not for H2.

In the final model step, incorporating the interaction between workforce composition and workforce-beneficiary contact, we observe significant changes (see Figures S1 and S2 in the online Supplemental Materials for a visualization of the interactions between representative workforce and interpersonal contact for each city).

In Vienna, a better match between the share of migrants among staff and the population, combined with strong workforce-beneficiary ties, is strongly associated with better diversity alignment (*b* = .859, *p* < .01). No such effect is observed with weak ties. This indicates that strong interpersonal ties, when combined with descriptive representation of the workforce, become the strongest predictor of diversity alignment of a nonprofit organization, surpassing the effect of descriptive representation alone. Introducing interaction effects neutralizes the direct effect of descriptive representation and the negative effect of weak workforce-beneficiary contact. Interestingly, formal representation regains its minimal but significant adverse effect, whereas public funding loses significance.

In Shenzhen, after introducing interaction terms, the direct effects of representative workforce and weak workforce-beneficiary contact on diversity alignment lose statistical significance. Unlike Vienna, the indirect effects of workforce-beneficiary contact do not significantly impact diversity alignment, possibly due to the small sample size. The subsequent section will interpret these findings in relation to existing theoretical discourse and the social and political contexts of both cities.

## Discussion

This study examines the complex relationship between nonprofit organizations and their diversifying environments, focusing on representative governance, interpersonal contact, and the influence of local state and market forces. Drawing on survey data on nonprofit organizations in Vienna and Shenzhen, our findings reveal that nonprofits with a workforce reflecting the migrant composition of their communities are more effective in reaching and serving migrants. Strong interpersonal ties between staff and beneficiaries are associated with a significant enhancement of diversity alignment in Vienna, especially when combined with a representative workforce, while weak ties have a negative association. Formal representation mechanisms do not relate positively to inclusion and are negatively associated with alignment in Shenzhen. These findings highlight the importance of workforce diversity and meaningful interpersonal interactions in enabling nonprofits to better respond to a diversifying environment, laying crucial groundwork for migrant inclusion.

The vision of cosmopolitan cities as inclusive melting pots remains fragile in both democratic and authoritarian regimes. Despite their multicultural urban tapestry, Vienna and Shenzhen still grapple with deep-rooted social divides. Contrary to idyllic depictions, urban civic spaces often fail to achieve true inclusion and diversity. Our study advances this discussion by examining the broader civic sectors and their roles in migrant inclusion and alienation, adding complexity to how civic spaces can influence these outcomes.

Do democratic practices in organizations improve inclusion and diversity? The answer is both yes and no. Our findings indicate that simply having a workforce reflective of community demographics and adopting formal electoral mechanisms in nonprofits do not automatically improve their diversity alignment as in the ability to include and serve migrants in proportion to their presence in the community. While democratic election of leaders is often assumed to enhance organizational responsiveness, our data suggest that formal representation may have little effect on diversity alignment efforts, potentially due to exclusionary and oligarchic tendencies in procedures or homophilic tendencies that reproduce dominant group interests within organizations (Pitkin, 1967; Weare et al., 2009).

Descriptive representation, however, does have a significant impact. Our findings support recent scholarship showing that an organization’s composition mirroring its community’s demographics can improve service to the community (e.g., Azevedo et al., 2021; Hale & Finchum-Mason, 2023). This is not merely a surface-level correlation. A workforce that reflects local migration diversity is more likely to possess the linguistic fluency, cultural familiarity, and social understanding needed to recognize and reduce structural and informal access barriers. These attributes can facilitate trust-building and enable organizations to anticipate the needs of migrant constituents more effectively. In our framework, we conceptualize workforce diversity as a causal mechanism that increases the likelihood of *diversity alignment*—defined here as the degree to which a nonprofit’s beneficiaries reflect the migrant composition of its surrounding community. Even under restrictive, authoritarian regimes, nonprofits with representative workforces can effectively address constituent needs, fostering democratic values and equitable public outcomes. Diversity alignment thus becomes an observable and measurable indicator of an organization’s capacity to include migrants fairly, even if it does not guarantee their long-term integration into society.

Yet, a representative workforce, while necessary, is not sufficient. Deep, personal interactions between staff and constituents enhance nonprofits’ responsiveness. Strong ties, characterized by in-depth knowledge of beneficiaries through regular, and meaningful interactions, amplify diversity alignment, especially when combined with a representative workforce. When such interpersonal engagement is present, organizations may be particularly well-positioned to co-create inclusive programs that not only reflect local demographic makeups but also lay the groundwork for mutual understanding across groups. This interactional dimension clarifies the pathway through which diversity alignment contributes to migrant inclusion as an intermediate organizational condition pushing toward migrant integration over time. Our research thus broadens the discourse in urban studies on how physical spaces influence intercultural contacts, which has largely focused on transient public encounters and their impact on social boundaries and negative attitudes (Blokland & Nast, 2014; Lobo, 2018; Peterson, 2017; Piekut & Valentine, 2017). By foregrounding nonprofits as semi-institutional sites shaped by sustained staff-beneficiary relationships, we point to new directions for understanding how everyday organizational practices can create conditions for migrant inclusion. Future research could build on this by examining how such nonprofit organizations might cultivate multicultural learning and foster positive emotional orientations toward migrants.

Nevertheless, not all contacts are beneficial, the nature and quality of interactions matter. Superficial contact, marked by limited personal knowledge from casual encounters, can negatively impact outreach and reinforce stereotypes. We align with emerging studies that question the assumption that intergroup contact necessarily reduces prejudice (Fincher et al., 2014; Kwan, 2013; Z. Liu et al., 2020). Irregular, less intensive interactions do not seem to stimulate sustained engagement among diverse groups, even when nonprofits implement formal democratic measures to improve representative capacity.

While not all nonprofits are obliged to respond to migrants, our study examines whether they adapt to a diversifying environment by becoming more inclusive, highlighting their potential as organizational resources for including migrants in their various primary focus areas. Our research shows that, despite relying on migrant labor, urban civic spaces in Vienna and Shenzhen fall short in supporting inclusion and nurturing migrants’ aspirations. In Vienna, linguistic and cultural hurdles seem to impede meaningful engagement, whereas in Shenzhen, institutional constraints are the primary obstacle. The Vienna government appears more proactive and effective in weaving migrants into the local societal fabric than Shenzhen, where authorities often view migrants as temporary labor solutions. This urban hostility hinders migrants from developing social and cultural capital for meaningful civic engagement in both contexts.

Our study has limitations, offering avenues for future research. Reliance on cross-sectional survey data at the organization-level limits our scope, and longitudinal studies would provide more comprehensive insights. Incorporating diverse data sources, such as qualitative interviews or document analysis, could enhance our findings. Finally, our focus on Vienna and Shenzhen, each with unique national political and institutional frameworks, means our insights are context-specific. Similar analyses in different geographic locations would help determine which trends hold across regions and which are context-bound.

## Conclusion

This study highlights the pivotal role nonprofit organizations can and do play in shaping inclusive civic spaces within diverse urban contexts. While representative workforces significantly enhance alignment with diversifying communities, especially when supported by strong interpersonal ties, formal democratic mechanisms alone are insufficient and may even hinder inclusion. Our findings challenge idealized notions of cosmopolitan cities by revealing persistent structural divides in both democratic and authoritarian regimes. Recognizing the importance of both descriptive representation and interpersonal contact, nonprofits hold untapped potential as spaces for intercultural engagement and social learning. Future research should build on these insights across varied political and urban settings, using longitudinal and mixed-method approaches to better capture the dynamics of migrant inclusion over time.

## Supplemental Material

sj-pdf-1-nvs-10.1177_08997640251387954 – Supplemental material for Bridging Divides or Widening Gaps? Nonprofit Organizations’ Efforts for Migrant Inclusion in Two Global Cities Supplemental material, sj-pdf-1-nvs-10.1177_08997640251387954 for Bridging Divides or Widening Gaps? Nonprofit Organizations’ Efforts for Migrant Inclusion in Two Global Cities by Yan Long, Wei Luo and Berta Terzieva in Nonprofit and Voluntary Sector Quarterly

## Notes

1. Pitkin (1967) distinguishes between formal, descriptive, substantive, and symbolic representation. Guo and Musso (2007) add a participatory dimension to Pitkin’s framework. We argue that to measure substantive and symbolic representation as well as participatory representation, the perceptions of beneficiaries are inevitable. Although intriguing, such exploration would go beyond the scope of this study, which emphasizes the organizational perspective. Therefore, we only look at descriptive and formal representation.

2. It was embedded in the regular city administration in 2004.

3. Note to Shenzhen: Acknowledging the potential for discrepancies in official records due to occasional inaccuracies and incompleteness, we dedicated significant effort to verifying the current operational status of registered nonprofit organizations in the list. If an organization lacks a valid address, telephone number, or any form of social media contact, we excluded it from our sample. To gain access to the randomly sampled nonprofit organizations and to test the validity of our survey instrument, we conducted pilot interviews with 48 experts in the field, including nonprofit sector professionals, academics, and government officials.

4. Note to Vienna: An additional sample was drawn, using the private business data provider *Herold Business Data LLC*. This second sample was purposefully targeted at nonprofit organizations with an annual budget of €25,000 or more, with the aim of oversampling larger organizations for specific analyses. The current study uses only the representative sample (i.e., not this additional sample of larger organizations) for Vienna, and all subsequent descriptions and analyses pertain to this first representative sample. A full description of the sampling methodology is published by Terzieva et al. (2024).
