# Supplementary material for: Bridging Divides or Widening Gaps? Nonprofit Organizations’ Efforts for Migrant Inclusion in Two Global Cities
Source: Nonprofit Volunt Sect Q. 2025 Nov 11;55(4):1049–74. doi: 10.1177/08997640251387954 (PMC13331349; doi:10.1177/08997640251387954)
Supplement: sj-pdf-1-nvs-10.1177_08997640251387954 – Supplemental material for Bridging Divides or Widening Gaps? Nonprofit Organizations’ Efforts for Migrant Inclusion in Two Global Cities [file sj-pdf-1-nvs-10.1177_08997640251387954.pdf]

Supplemental Materials for

Bridging Divides or Widening Gaps? Nonprofit Organizations' Efforts for Migrant Inclusion in Two Global Cities

Table S1. Sample Descriptives (I): Field of Activity

	Vienna	Shenzhen
ICNPO Categories		
Culture and recreation	45.0%	24.1%
Education and research	10.1%	7.9%
Health	4.7%	3.4%
Social services	11.5%	21.7%
Environment	3.6%	3.0%
Development and housing	6.7%	6.9%
Law, advocacy and politics	4.5%	3.0%
Philanthropic intermediaries and voluntarism promotion	0.0%	3.9%
International	2.0%	0.0%
Religion	1.7%	1.5%
Business and professional associations	9.8%	21.7%
Other	0.6%	3.0%
Total N	358	203

Table S2. Sample Descriptives (II): NPO Characteristics (Shenzhen)

	Valid N	Min	Max	Mean	S.D.
Share of migrant beneficiaries	140	0.00	1.00	0.43	0.29
Share of migrant workforce	195	0.00	1.00	0.38	0.30
Share of migrants in local population	203	0.14	0.99	0.70	0.12
Organizational age, in years	197	2	31	7.42	6.26
Share of nonprofit organizations with members	203	0	1	0.60	0.49

Table S3. Sample Descriptives (II): NPO Characteristics (Vienna)

	Valid N	Min	Max	Mean	S.D.
Share of migrant beneficiaries	331	0.00	1.00	0.18	0.24
Share of migrant workforce	344	0.00	1.00	0.10	0.19
Share of migrants in local population	358	0.06	0.45	0.24	0.10
Organizational age, in years	358	3.00	99.00	32.08	23.28
Share of nonprofit organizations with members	356	0.00	1.00	0.87	0.33

Table S4. Correlation Table Vienna

		1	2	3	4	5	6	7	8	9	10
1	diversity alignment	1.000									
2	descriptive representation of workforce	0.585	1.000								
3	formal representation	-0.129	0.002	1.000							
4	weak workforce-beneficiary contact	-0.120	0.024	0.266	1.000						
5	strong workforce-beneficiary contact	-0.077	0.031	0.289	0.604	1.000					
6	public funding	0.156	0.043	-0.067	0.043	-0.123	1.000				
7	private funding	0.074	0.004	-0.062	-0.063	-0.034	-0.202	1.000			
8	organizational size	0.071	-0.069	-0.183	0.018	-0.086	0.223	0.084	1.000		
9	membership organization	-0.233	-0.148	0.184	0.062	0.158	-0.175	-0.109	-0.103	1.000	
10	field of activity	-0.024	0.012	-0.056	-0.240	-0.238	0.118	-0.086	0.041	-0.016	1.000

Table S5. Correlation Table Shenzhen

		1	2	3	4	5	6	7	8	9	10
1	diversity alignment	1.000									
2	descriptive representation of workforce	0.356	1.000								
3	formal representation	-0.215	-0.093	1.000							
4	weak workforce-beneficiary contact	-0.214	0.122	0.087	1.000						
5	strong workforce-beneficiary contact	-0.136	0.081	0.090	0.597	1.000					
6	public funding	0.049	0.030	-0.186	-0.056	-0.091	1.000				
7	private funding	0.201	0.199	-0.115	0.110	-0.043	-0.278	1.000			
8	organizational size	-0.011	-0.081	-0.071	0.080	0.061	0.016	0.190	1.000		
9	membership organization	-0.195	-0.230	0.442	0.113	0.273	-0.110	-0.337	-0.172	1.000	
10	field of activity	-0.018	-0.035	0.146	-0.021	-0.054	-0.112	-0.043	0.259	0.054	1.000

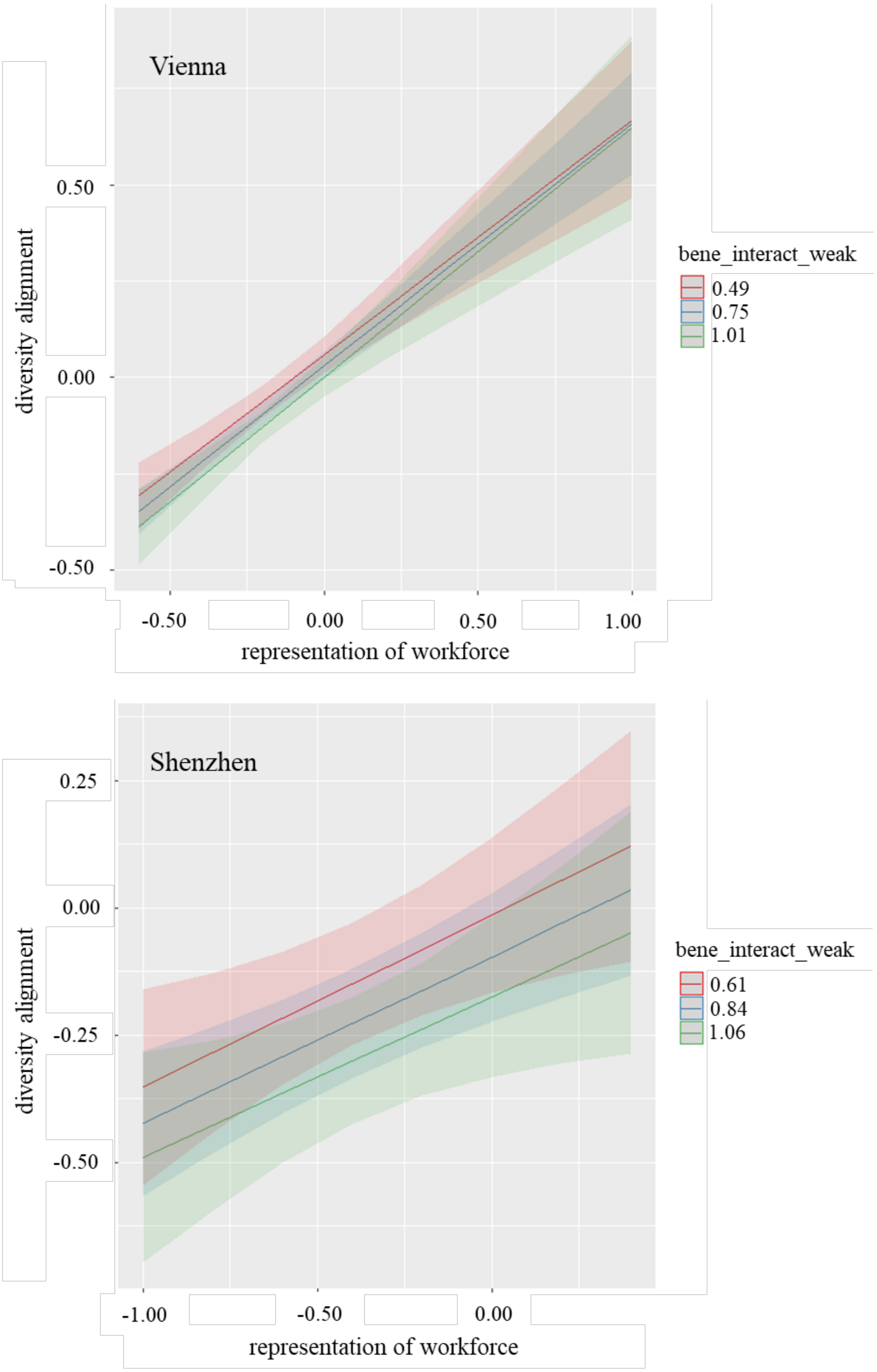

Figure S1. Predicted values of diversity alignment by descriptive representation of workforce and weak workforce-beneficiary contact

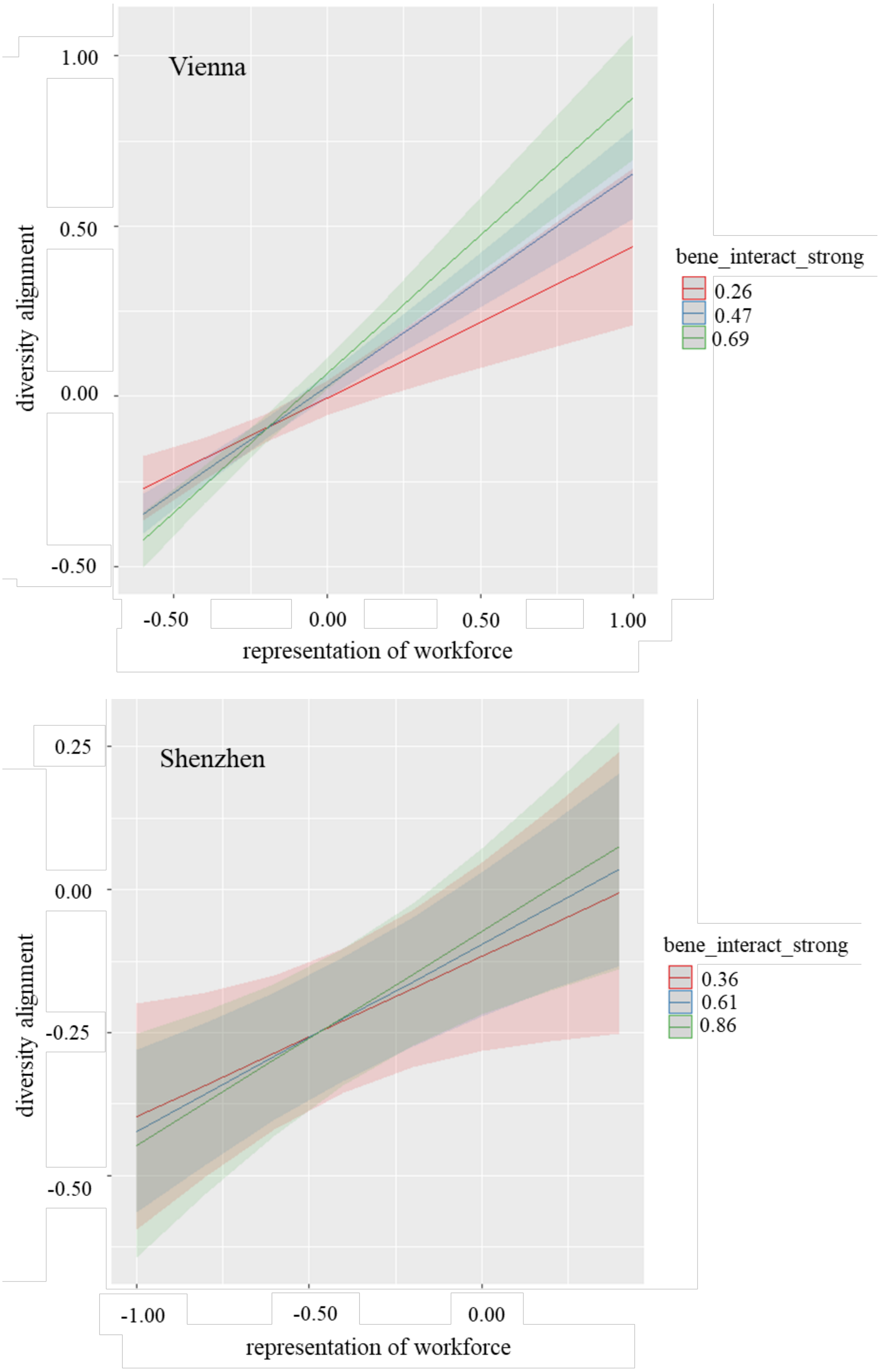

Figure S2. Predicted values of diversity alignment by descriptive representation of workforce and strong workforce-beneficiary contact
